# A Unique Case of Anomalous Origin of the Left Main Coronary Artery From the Right Coronary Sinus

**DOI:** 10.7759/cureus.105187

**Published:** 2026-03-13

**Authors:** Justin Rouintan, Saadeh Saadeh

**Affiliations:** 1 Family Medicine, Kingman Regional Medical Center, Kingman, USA; 2 Cardiology, Kingman Reigonal Medical Center, Kingman, USA

**Keywords:** anomalous, anomalous left main coronary artery, cardiac sudden death, cardiology, congenital defect, coronary artery, coronary ostium, coronary sinus ostium, right coronary artery (rca), single ostium coronary artery

## Abstract

Anomalous origination and course of a coronary artery is a rare congenital finding, with clinical significance that varies depending on the vessel's anatomic trajectory. The risk of sudden cardiac death in affected patients is unpredictable and is largely determined by the relationship of the anomalous vessel to the great arteries. Anomalous origin of the left main coronary artery (LMCA) off the right sinus of Valsalva (RSV) is associated with increased risk of cardiac events, particularly when the vessel follows an inter-arterial or intramural course, which may result in dynamic compression resulting in myocardial ischemia. Here we present a case of a 63-year-old female with exertional chest pain and dyspnea whose initial non-invasive cardiac workup was unremarkable. Subsequent coronary angiography demonstrated an LMCA that arose from a shared ostium with the right coronary artery (RCA) in the RSV. Coronary computed tomography angiography (CTA) revealed an anterior and superior course relative to the great vessels. Given the benign anatomic course, the patient was managed conservatively without surgical intervention.

## Introduction

Anomalous origin of the LMCA from the RSV is a rare congenital coronary anomaly that is infrequently identified in older adults [1-3]. This anomaly is typically detected earlier in life due to its association with myocardial ischemia, malignant arrhythmias, and sudden cardiac death [1,4-6]. Prior studies have reported that up to 59% of patients with anomalous LMCA experience sudden cardiac death before the age of 20, highlighting the potentially lethal nature of this condition [1]. Prior to diagnosis, patients may present with angina pectoris, exertional dyspnea, myocardial infarction, heart failure, dysrhythmias, or sudden cardiac arrest, although many remain asymptomatic until a catastrophic event occurs [1,4-6].

The risk of sudden cardiac death in patients with anomalous LMCA depends largely on the vessel’s anatomic course in relation to the aorta and pulmonary artery [4-7]. The reported incidence of LMCA arising from the RSV is approximately 0.15% in patients undergoing coronary angiography [2,3]. Anomalous LMCA variants are classified according to their relationship with the great vessels and include inter-arterial, intramyocardial (septal), posterior (retro-aortic), and anterior courses [4]. The greatest risk of ischemic events is associated with an inter-arterial course, in which the LMCA travels between the aorta and pulmonary artery, often with a concomitant intramural segment that predisposes the vessel to systolic compression [4,5]. Intra-myocardial courses, in which the LMCA travels through the sub-endocardial or intramyocardial region of the ascending aorta, are also associated with increased risk. Anterior and posterior courses are generally considered lower-risk variants [4-7].

Current expert consensus guidelines recommend surgical intervention for patients with anomalous coronary arteries that demonstrate high-risk anatomy, particularly when both coronary arteries arise from the same sinus or from a single ostium, as these configurations are associated with adverse clinical outcomes [6]. We report a case of anomalous LMCA arising from the RSV with a shared ostium and a benign anterior course, diagnosed late in life and successfully managed conservatively.

## Case presentation

A 63-year-old female presented for evaluation of retrosternal chest pain and exertional dyspnea. Two to three weeks prior to presentation, she developed a burning, left-sided retrosternal chest pain radiating to her left arm, shoulders, and jaw, accompanied by new-onset shortness of breath with exertion. Her medical history was significant for colon cancer, hypertension, hyperlipidemia, rheumatoid arthritis, migraines, vertigo, and orthostatic dizziness. Home medications include hydrocodone, fenofibrate, fluticasone, rizatriptan, ropinirole, clonidine, erenumab, and low-dose baby aspirin. She was a former smoker with a five-pack-year history and had a family history notable for premature coronary artery disease in a grandmother and uncle.

**Figure 1:**
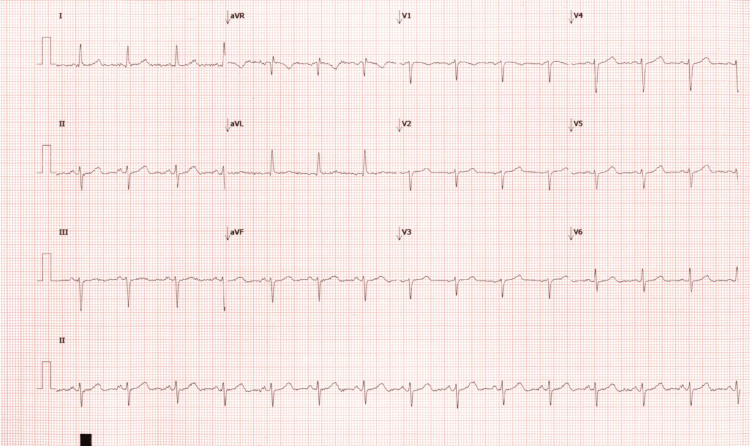
EKG Patients EKG that was completed when symptoms were present. Showing sinus rhythm with heart rate of 88 BPM, left axis deviation, and PR interval of 126ms, QRS of 84ms, QTc of 455ms. No significant ST changes or concerning abnormalities.

Initial evaluation included electrocardiography (ECG), ambulatory event monitoring, carotid doppler ultrasonography, transthoracic echocardiography, and nuclear stress testing. The ECG showed no ischemic changes or significant abnormalities (Figure 1). Carotid Doppler imaging demonstrated mild atheromatous disease at the carotid bifurcations without hemodynamically significant stenosis. Nuclear stress testing revealed no evidence of inducible ischemia or prior infarction. Transthoracic echocardiography demonstrated a mildly reduced left ventricular ejection fraction of 40-45% without significant valvular abnormalities.

**Figure 2:**
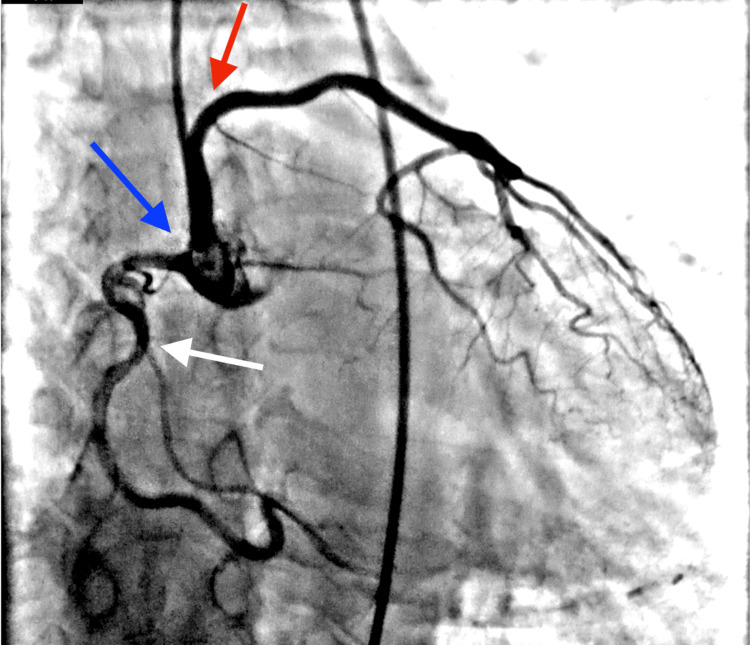
Left heart catheterization Left heart catheterization demonstrating anomalous origin of the left main coronary artery (LMCA). The LMCA (red arrow) arises from the right sinus of Valsalva (RSV) (blue arrow) via a shared ostium with the right coronary artery (RCA) (white arrow). The image highlights the single coronary ostial origin within the RSV, consistent with anomalous LMCA arising from the opposite sinus.

**Figure 3:**
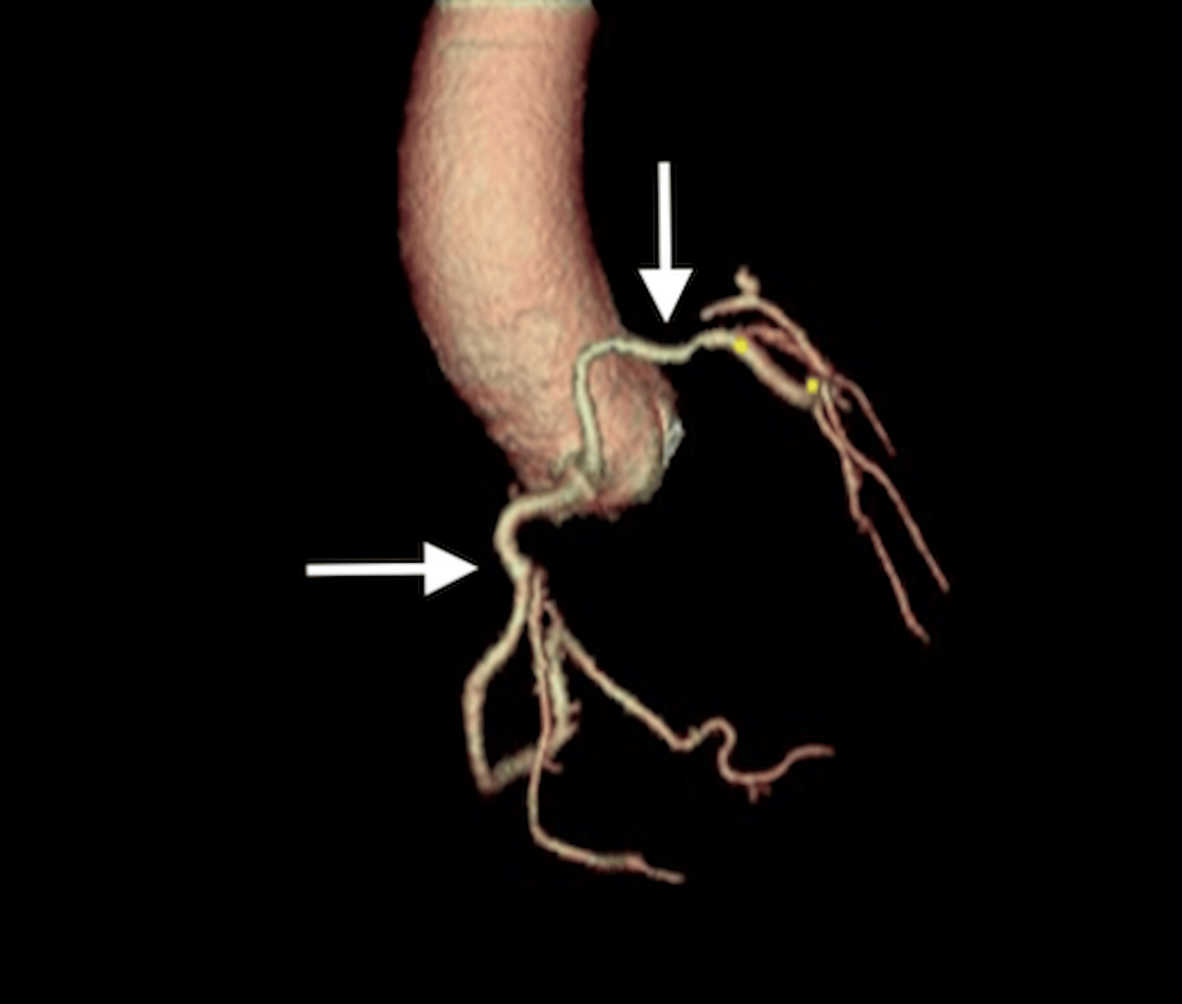
3D coronary computed tomography angiography Three-dimensional coronary computed tomography angiography (CTA) demonstrating anomalous origin of the left main coronary artery (LMCA). The LMCA (vertical arrow) arises from the right sinus of Valsalva (RSV) via a shared ostium with the right coronary artery (RCA) (horizontal arrow). The 3D reconstruction clearly depicts the single coronary origin from the RSV prior to the bifurcation of the LMCA into its major branches.

**Figure 4:**
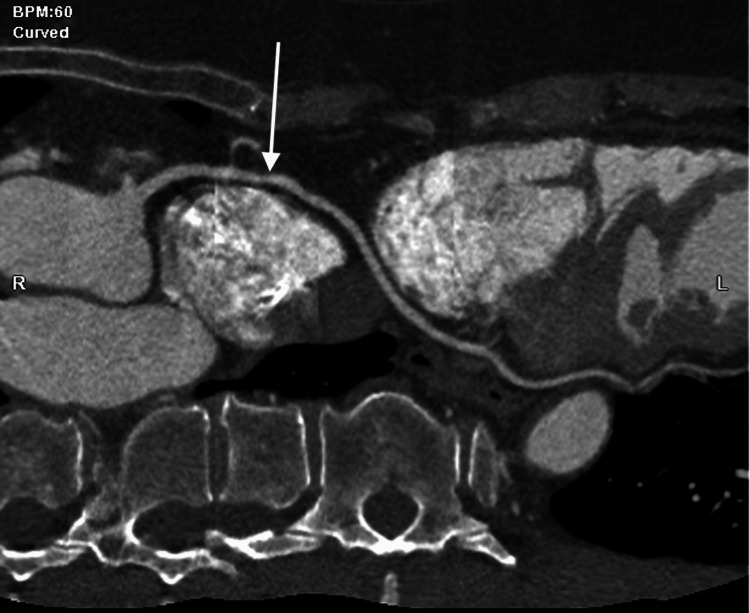
2D curved multiplanar reconstruction Two-dimensional curved multiplanar reconstruction from coronary computed tomography angiography (CTA) demonstrating a large anomalous left main coronary artery (LMCA) (white arrow) arising from the right coronary ostium. The vessel is shown coursing superiorly and anteriorly to the pulmonary artery, consistent with a low-risk anterior trajectory without evidence of an inter-arterial or intramural course.

**Figure 5:**
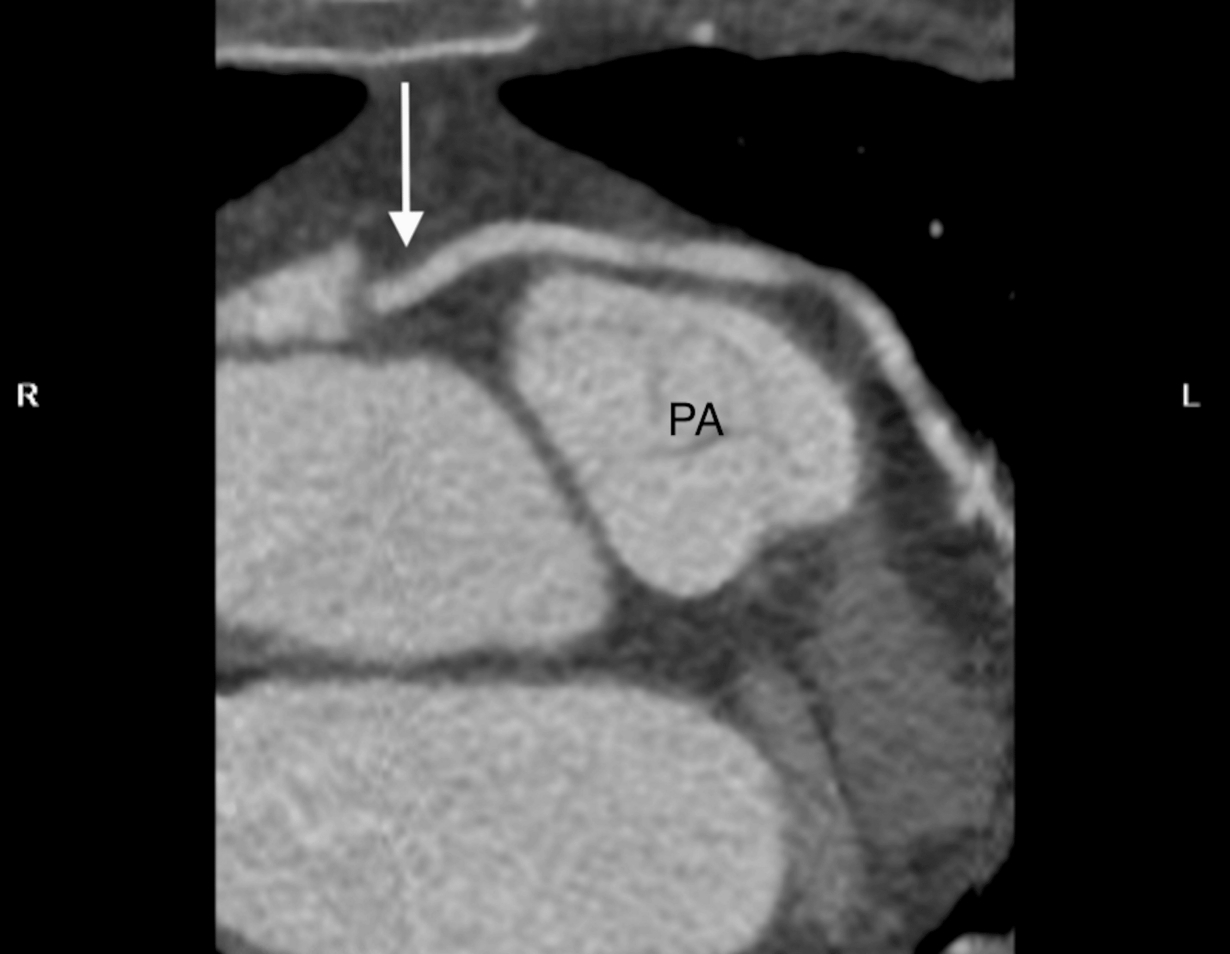
2D coronary CTA Two-dimensional coronary computed tomography angiography (CTA) showing the anomalous large left main coronary artery (LMCA) (white arrow) arising from the right sinus of Valsalva (RSV) and coursing superiorly and anteriorly to the pulmonary artery (PA). The image highlights the vessel’s low-risk anterior course and relationship to the great vessels, supporting conservative management.

Given persistent symptoms despite noninvasive testing, the patient underwent left heart catheterization, which demonstrated an anomalous origin of the LMCA from the RSV (Figure 2). Apparent systolic compression of the anomalous vessel raised concern for a potentially malignant inter-arterial course, prompting further evaluation with coronary CTA. CTA confirmed a dominant, widely patent RCA arising from a shared ostium with the LMCA and demonstrated that the LMCA coursed superiorly and anterior to the pulmonary artery, supplying the left anterior descending and circumflex arteries (Figures 3-5). These findings were consistent with a low-risk anterior variant of anomalous LMCA.

The case was reviewed by multiple cardiothoracic surgeons, all of whom determined that the anomaly was stable and not amenable to surgical intervention. The patient has since been followed closely by cardiology with serial monitoring and has experienced no recurrent symptoms or adverse cardiac events.

## Discussion

Most coronary artery anomalies are clinically benign; however, coronary arteries arising from the opposite sinus of Valsalva are associated with substantially increased risk of myocardial ischemia and sudden cardiac death, particularly when the anomalous vessel courses between or adjacent to the great vessels [4-7]. In this case, the LMCA followed an anterior and superior course relative to the aorta and pulmonary artery, a configuration that is generally considered low risk and appropriate for conservative management [4-7].

The global prevalence of coronary artery anomalies has been reported to be approximately 5.64%, with anomalous origin of the RCA from the left sinus occurring in 0.92% of cases and anomalous origin of the LMCA from the right sinus occurring in approximately 0.15% of cases [2,3]. The exact mechanism of sudden cardiac death in relation to coronary anomalies is not well understood. However, based on anatomic and physiologic variables in the origination and course of the anomalous artery, certain factors seem to predispose to ischemic events and lethal ventricular arrhythmias. Possible mechanisms include vessel spasm, oblique take-off from the aorta, ostial ridge, intussusception, noncompliant pericommissural area, and finally, dynamic compression of the anomalous coronary artery intramurally and/or between the great arteries. [6]. These mechanisms are thought to predispose patients to myocardial ischemia and malignant ventricular arrhythmias, particularly during periods of increased cardiac demand [6,8].

Diagnosis of anomalous coronary arteries often occurs only after the onset of symptoms, as fewer than 20% of patients present with warning symptoms prior to a catastrophic event, and sudden cardiac death may be the initial presentation [8]. While coronary angiography remains the diagnostic gold standard, coronary CTA plays a critical role in accurately defining the vessel’s course and identifying intramural or inter-arterial components that influence management decisions [6-8]. In this patient, persistent exertional symptoms despite normal stress testing appropriately prompted invasive angiography and subsequent CTA, leading to a definitive diagnosis.

Surgical intervention is recommended for symptomatic patients with high risk-coronary anatomy, whereas management of asymptomatic or low-risk variants remains controversial and should be individualized based on anatomic findings and clinical presentation [6-8]. Surgical options include unroofing of an intramural segment, pulmonary artery translocation, ostial reimplantation, creation of a neo-ostium, and coronary artery bypass grafting [6-8]. Unroofing is generally preferred when an intramural course is present, while reimplantation or anatomic repair may be favored in the absence of intramural involvement [8]. In the present case, surgical intervention was not indicated due to the absence of an inter-arterial or intramural course, and the patient has remained clinically stable with conservative management.

## Conclusions

Anomalous origin of the LMCA from the RSV is a rare but potentially life-threatening congenital coronary anomaly that warrants careful anatomic characterization and multidisciplinary evaluation due to its association with sudden cardiac death. This case is notable for a shared ostium of the LMCA and RCA with a benign anterior and superior course, late diagnosis in adulthood, and successful conservative management without surgical intervention. Accurate delineation of coronary anatomy allowed for appropriate risk stratification and avoidance of unnecessary surgery.

## References

[1] Angelini P, Velasco JA, Flamm S (2002). Coronary anomalies: Incidence, pathophysiology, and clinical relevance. Circulation.

[2] Angelini P (2007). Coronary artery anomalies: An entity in search of an identity. Circulation.

[3] Yamanaka O, Hobbs RE (1990). Coronary artery anomalies in 126,595 patients undergoing coronary arteriography. Cathet Cardiovasc Diagn.

[4] Ropers D, Gehling G, Pohle K (2002). Anomalous course of the left main or left anterior descending coronary artery originating from the right sinus of valsalva: identification of four common variations by electron beam tomography. Circulation.

[5] Frommelt PC, Frommelt MA, Tweddell JS, Jaquiss RDB (2003). Prospective echocardiographic diagnosis and surgical repair of anomalous origin of a coronary artery from the opposite sinus with an interarterial course. J Am Coll Cardiol.

[6] Brothers JA, Frommelt MA, Jaquiss RD, Myerburg RJ, Fraser CD Jr, Tweddell JS (2017). Expert consensus guidelines: Anomalous aortic origin of a coronary artery. J Thorac Cardiovasc Surg.

[7] Tuncer C, Batyraliev T, Yilmaz R, Gokce M, Eryonucu B, Koroglu S (2006). Origin and distribution anomalies of the left anterior descending artery in 70,850 adult patients: Multicenter data collection. Catheter Cardiovasc Interv.

[8] Khan MS, Idris O, Shah J, Sharma R, Singh H (2020). Anomalous origin of left main coronary artery from the right sinus of Valsalva: A case series-based review. Cureus.

